# Effects of ozone water irrigation and spraying on physiological characteristics and gene expression of tomato seedlings

**DOI:** 10.1038/s41438-021-00618-8

**Published:** 2021-09-01

**Authors:** Jin-Peng Xu, Yan-Chong Yu, Tao Zhang, Qian Ma, Hong-Bing Yang

**Affiliations:** Key Lab of Plant Biotechnology in Universities of Shandong Province, College of Life Sciences, Qingdao Agricultural University, 266109 Qingdao, China

**Keywords:** Plant physiology, Plant molecular biology

## Abstract

Tomato seedlings were used as experimental materials and treated with 1.0, 2.0, 3.0, and 4.0 mg/L ozone water irrigation and 0.2, 0.4, 0.6, and 0.8 mg/L ozone water spray treatments. Indexes including the malondialdehyde (MDA) content, superoxide dismutase (SOD), peroxidase (POD) and catalase (CAT), activities, soil and plant analysis development (SPAD) value, and nitrogen content of leaves were measured. Furthermore, the expression of antioxidant enzyme, chlorophyll synthesis and nitrogen absorption genes was analyzed after optimal ozone water treatment. The results showed that the activities of antioxidant enzymes in tomato leaves were significantly increased, and the MDA content in tomato leaves was significantly reduced by ozone water irrigation and spray treatment, which indicated that ozone water treatment can significantly improve the stress tolerance of tomato seedlings. Ozone water irrigation and spraying could also significantly increase the leaf SPAD value and nitrogen content of tomato seedlings, and the optimal concentrations of ozone water irrigation and spraying were 3.0 mg/L and 0.6 mg/L, respectively. The effect of ozone water irrigation on improving the physiological characteristics of tomato seedlings was better than that of spraying. After treatment with the optimal concentration of ozone water, the relative expression of antioxidant enzyme, chlorophyll synthesis, and nitrogen absorption genes was significantly increased, and the maximum expression level was reached at 12 h. In addition, ozone water irrigation could promote the expression of genes more than ozone water spraying, which was consistent with the improvements in the physiological characteristics of the tomato seedlings.

## Introduction

Tomato (*Solanum lycopersicum*) is an annual herb in the Solanaceae. Tomato is mainly divided into two complex populations: common tomato and Peruvian tomato. The cultivated varieties used in agricultural production are common tomatoes. Tomato is sweet and sour, with excellent taste. It contains vitamins, carotene, organic acids, and minerals. Because of its high edibility and rich nutritional value, it has become one of the most common fruits and vegetables cultivated worldwide, and the cultivation area is still expanding^1^. Studies have shown that the lycopene in tomato has a strong antioxidant effect, and lycopene supplementation can resist aging, enhance immunity and reduce disease occurrence, indicating that lycopene is of important health care value^2^. In recent years, studies have shown that diseases and insect pests, poor flower bud differentiation, and fruit cracking have become the main problems in tomato production^3^. With the modern increase in environmental protection awareness, treatment methods for diseases and insect pests are becoming more restrictive. However, the high intensity use pesticides is still the preferred control method for fruit growers^4^. The extensive use of pesticides not only pollutes the air, soil, and water but also results in residues in fruits and vegetables, endangering human health^5^. Therefore, green pest prevention and control methods have become a key link in agricultural production.

As a natural strong oxidant and bactericide, ozone has the characteristics of a broad spectrum of activity, high efficiency, and no residue. It has been widely used in industry, medicine, agriculture, and other fields. According to research, as a clean oxidant without secondary pollution, ozone can be used in industrial wastewater treatment^6^. Cheng et al. (2015)^7^ found that medical ozone has a protective effect against brain injury in zebrafish juveniles with hypoxic brain damage. Liu et al. (2019)^8^ revealed that ozone treatment delayed the senescence of *Flammulina velutipes* and was beneficial to its preservation. Zhou et al. (2012)^9^ explained that ozone could effectively improve the degradation rate of chlorothalonil in soil. Zhang et al. (2019)^10^ treated tomato and cucumber seeds with ozone for 1 year and found that microbial growth on the surface of seeds was effectively inhibited, and the germination rate and vigor of seeds were improved. Previous studies have reported that ozone has outstanding advantages in insecticides, sterilization, fresh storage, and oxidation, but many studies have noted that ozone has certain toxic effects on plant growth and development^11^. The toxic effect of ozone on plants has a common feature: it can result in local cell death^12^. When the concentration of ozone was as high as 98 g/L, the leaves of trees and shrubs were damaged by ozone^13^. However, low-concentration ozone water treatment could decompose the pesticide residues in soil^14^, prevent the occurrence of tomato bacterial wilt^15^ and root-knot nematodes of ginger^16^, and increase crop quality and yield. These studies showed that the effect of ozone water is better in prevention and control than that of pure ozone gas. Although ozone has broad application prospects in agricultural production^17^, the effects of ozone water on plant physiological characteristics and gene expression are still largely unclear. In this paper, the effects of ozone water irrigation and spraying on the physiological characteristics of tomato seedlings were compared by measuring the MDA content, activities of antioxidant enzymes (SOD, POD, and CAT), SPAD value and nitrogen (N) content. By analyzing the expression levels of antioxidant enzyme genes, chlorophyll synthesis-related genes, and N absorption-related genes, the effects of ozone water treatment on plants were further revealed at the molecular level, providing a basis for the efficient utilization of ozone water in agriculture.

## Results

### Effects of ozone water irrigation and spraying on the MDA content of tomato leaves

**Fig. 1:**
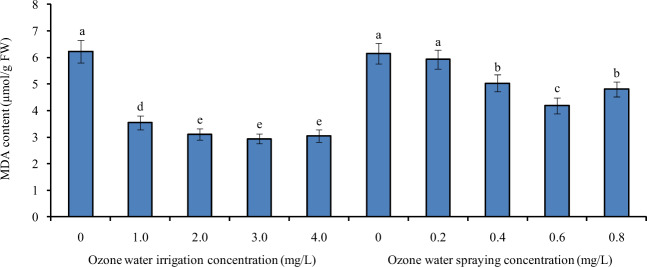
The MDA content of tomato leaves under different concentrations of ozone water irrigation and spraying. Error bars represent the SD (*n* = 3). Values followed by different letters are significantly different according to Tukey’s test (*P* < 0.05)

Figure 1 shows the MDA content of tomato leaves under different concentrations of ozone water irrigation and spray treatment. After 1.0, 2.0, 3.0, and 4.0 mg/L ozone water irrigation, the MDA content of tomato leaves was significantly decreased by 43%, 50%, 53%, and 51% compared with the control level, and the largest decrease was found under the 3.0 mg/L ozone water irrigation. After spraying 0.2 mg/L ozone water, there was no significant difference in the MDA content of tomato leaves compared with the control, indicating that the effect of low-concentration ozone water on the MDA content of tomato leaves was small. However, after spraying 0.4, 0.6, and 0.8 mg/L ozone water, the MDA content of tomato leaves was significantly decreased by 18%, 32%, and 22% compared with the control, and spraying 0.6 mg/L ozone water resulted in the greatest decrease. Compared with ozone water spraying, ozone water irrigation had a better effect on reducing the MDA content of tomato leaves.

### Effects of ozone water irrigation and spraying on the SOD activity in tomato leaves

**Fig. 2:**
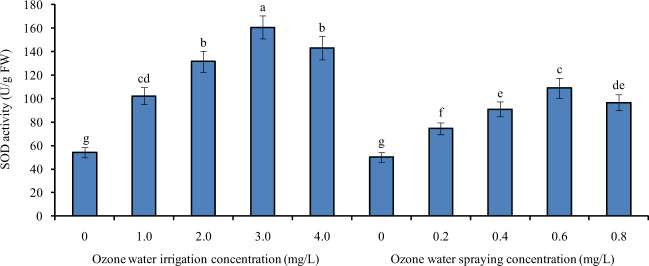
The SOD activity of tomato leaves under different concentrations of ozone water irrigation and spraying. Error bars represent the SD (*n* = 3). Values followed by different letters are significantly different according to Tukey’s test (*P* < 0.05)

Figure 2 shows the SOD activity of tomato leaves under different concentrations of ozone water irrigation and spraying. After 1.0, 2.0, 3.0, and 4.0 mg/L ozone water irrigation, the SOD activity of tomato leaves was significantly increased by 89%, 143%, 197%, and 165% compared with the control level, and the largest increase was found with 3.0 mg/L ozone irrigation. After spraying 0.2, 0.4, 0.6, and 0.8 mg/L ozone water, the SOD activity of tomato leaves was also significantly increased by 49%, 82%, 117%, and 93% compared with the control, and spraying 0.6 mg/L ozone water resulted in the greatest increase. The effect of ozone water irrigation on improving the SOD activity of tomato leaves was better than that of ozone water spraying.

### Effects of ozone water irrigation and spraying on the POD activity of tomato leaves

**Fig. 3:**
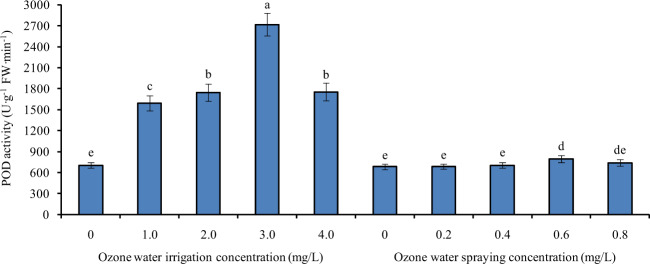
The POD activity of tomato leaves under different concentrations of ozone water irrigation and spraying. Error bars represent the SD (*n* = 3). Values followed by different letters are significantly different according to Tukey’s test (*P* < 0.05)

Figure 3 shows the POD activity of tomato leaves under different concentrations of ozone water irrigation and spraying. After 1.0, 2.0, 3.0, and 4.0 mg/L ozone water irrigation, the POD activity of tomato leaves was significantly increased by 126%, 147%, 286%, and 149% compared with the control level, and the largest increase was found under 3.0 mg/L ozone irrigation. After spraying 0.2, 0.4, and 0.8 mg/L ozone water, the POD activity of tomato leaves increased slightly, and it was not significantly different from that of the control. After spraying 0.6 mg/L ozone water, the POD activity of tomato leaves was significantly increased by 16% compared with that of the control. The effect of ozone water irrigation on improving the POD activity of tomato leaves was better than that of ozone water spraying.

### Effects of ozone water irrigation and spraying on the CAT activity of tomato leaves

**Fig. 4:**
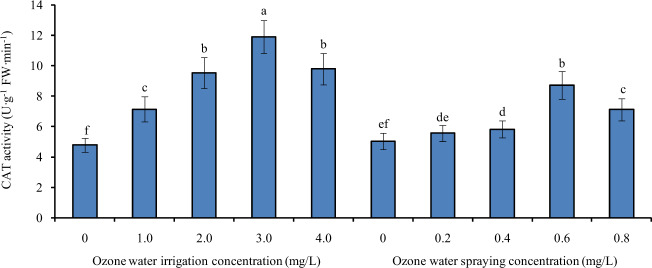
The CAT activity of tomato leaves under different concentrations of ozone water irrigation and spraying. Error bars represent the SD (*n* = 3). Values followed by different letters are significantly different according to Tukey’s test (*P* < 0.05)

Figure 4 shows the CAT activity of tomato leaves under different concentrations of ozone water irrigation and spraying. After 1.0, 2.0, 3.0, and 4.0 mg/L ozone water irrigation, the CAT activity of tomato leaves was significantly increased by 49%, 99%, 149%, and 105% compared with that of the control, and the largest increase was found with 3.0 mg/L ozone irrigation. After spraying 0.2 mg/L ozone water, there was no significant difference in the CAT activity of tomato leaves compared with the control, indicating that the effect of low-concentration ozone water on the CAT activity of tomato leaves was small. However, after spraying 0.4, 0.6, and 0.8 mg/L ozone water, the CAT activity of tomato leaves was significantly increased by 16%, 74%, and 42% compared with the control level, and spraying 0.6 mg/L ozone water resulted in the greatest increase. Compared with ozone water spraying, ozone water irrigation had a better effect on improving the CAT activity of tomato leaves.

### Effects of ozone water irrigation and spraying on the SPAD value of tomato leaves

**Fig. 5:**
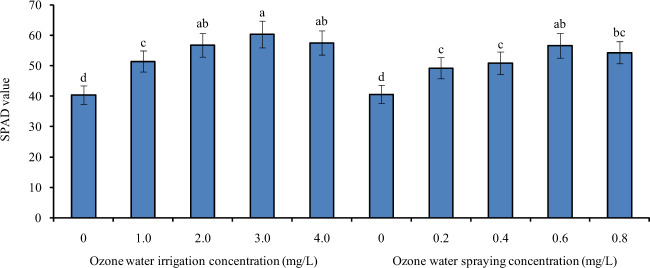
The SPAD values of tomato leaves under different concentrations of ozone water irrigation and spraying. Error bars represent the SD (*n* = 3). Values followed by different letters are significantly different according to Tukey’s test (*P* < 0.05)

Figure 5 shows the SPAD values of tomato leaves under different concentrations of ozone water irrigation and spraying. The SPAD value of tomato leaves was significantly increased by 27%, 41%, 50%, and 43% after 1.0, 2.0, 3.0, and 4.0 mg/L ozone water irrigation, and the largest increase was found with the 3.0 mg/L ozone water treatment. After spraying 0.2, 0.4, 0.6, and 0.8 mg/L ozone water, the SPAD value of tomato leaves was also significantly increased by 21%, 25%, 39%, and 34% compared with that of the control, and spraying 0.6 mg/L ozone water resulted in the greatest increase. Ozone water irrigation had a better effect on improving the SPAD value of tomato leaves than did ozone water spraying.

### Effects of ozone water irrigation and spraying on the N content of tomato leaves

**Fig. 6:**
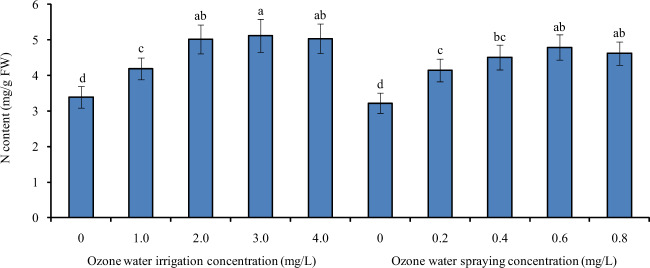
The N contents of tomato leaves under different concentrations of ozone water irrigation and spraying. Error bars represent the SD (*n* = 3). Values followed by different letters are significantly different according to Tukey’s test (*P* < 0.05)

Figure 6 shows the N contents of tomato leaves under different concentrations of ozone water irrigation and spraying. After 1.0, 2.0, 3.0, and 4.0 mg/L ozone water irrigation, the N content of tomato leaves was significantly increased by 24%, 48%, 51%, and 49% compared with that of the control, and the largest increase was found with the 3.0 mg/L ozone water treatment. After spraying 0.2, 0.4, 0.6, and 0.8 mg/L ozone water, the N content of tomato leaves was also significantly increased by 29%, 40%, 49%, and 43% compared with the control, and spraying 0.6 mg/L ozone water resulted in the greatest increase. The effect of ozone water irrigation on improving the N content of tomato leaves was slightly better than that of ozone water spraying.

### Expression profiles of antioxidant enzyme genes in tomato leaves under optimal concentrations of ozone water irrigation and spraying

**Fig. 7:**
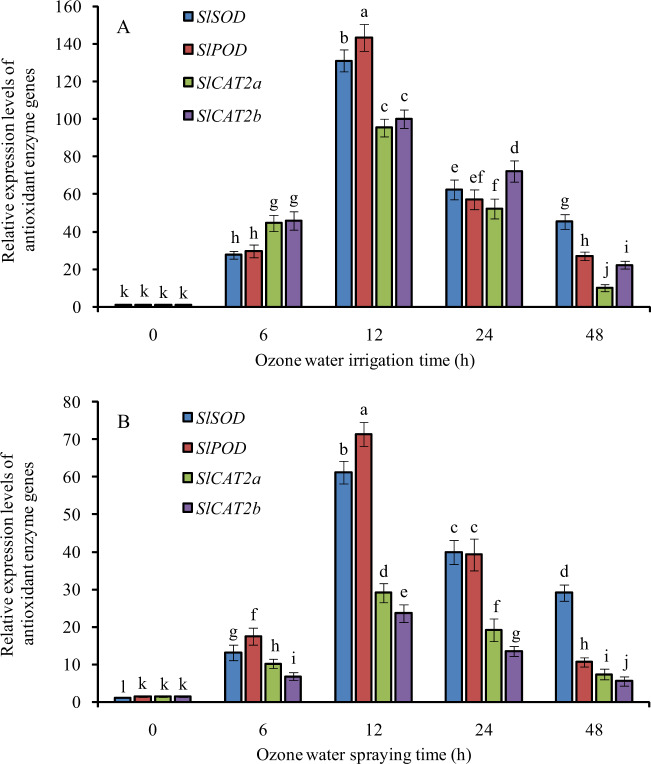
Relative expression of antioxidant enzyme genes at 0, 6, 12, 24, and 48 h after ozone water treatments. **A** ozone water irrigation, **B** ozone water spraying. Error bars represent the SD (*n* = 3). Values followed by different letters are significantly different according to Tukey’s test (*P* < 0.05)

After 3.0 mg/L ozone water irrigation, the MDA content of tomato leaves decreased the most, and the activities of SOD, POD, and CAT, the SPAD value and nitrogen content of tomato leaves increased the most. Therefore, the optimal concentration of ozone water irrigation was 3.0 mg/L. The relative expression levels of *SlSOD*, *SlPOD*, *SlCAT2a*, and *SlCAT2b* in tomato leaves were significantly increased after the optimal concentration of ozone water irrigation and reached the maximum expression level at 12 h after treatment, with levels increased 130-, 142-, 82-, and 104-fold compared with the control. Among them, *SlPOD* was increased the most, followed by *SlSOD*, *SlCAT2b*, and *SlCAT2a* (Fig. 7A).

After spraying 0.6 mg/L ozone water, the MDA content of tomato leaves decreased the most, and the activities of SOD, POD, and CAT, the SPAD value and nitrogen content of tomato leaves increased the most. Therefore, the optimal concentration of ozone water spray treatment was 0.6 mg/L. The relative expression levels of *SlSOD*, *SlPOD*, *SlCAT2a*, and *SlCAT2b* in tomato leaves were significantly increased after optimal concentrations of ozone water spraying and reached the maximum expression level at 12 h after treatment, with levels increased 60-, 48-, 20-, and 16-fold compared with the control. Among them, *SlSOD* was increased the most, followed by *SlPOD*, *SlCAT2a*, and *SlCAT2b* (Fig. 7B).

### Expression profiles of chlorophyll synthesis-related genes in tomato leaves under optimal concentrations of ozone water irrigation and spraying

**Fig. 8:**
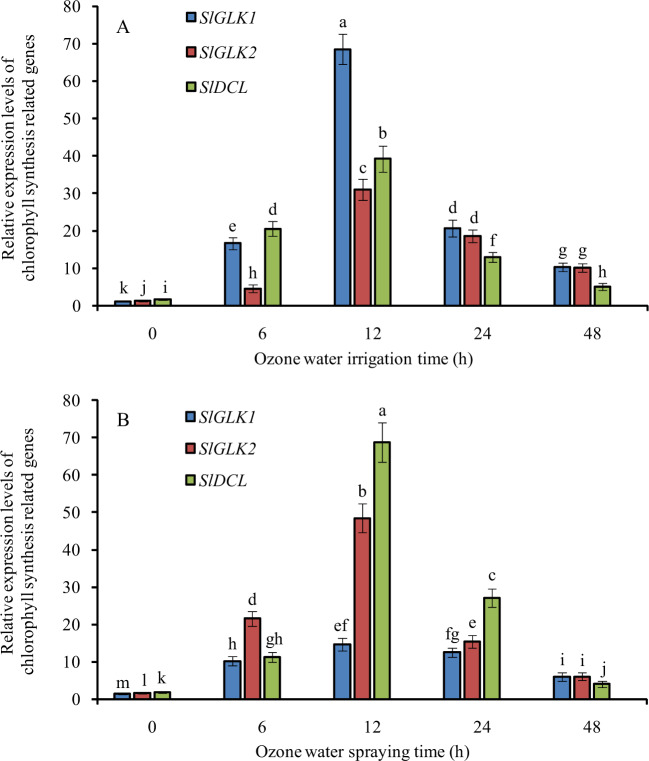
Relative expression of chlorophyll synthesis-related genes at 0, 6, 12, 24, and 48 h after ozone water treatments. **A** ozone water irrigation, **B** ozone water spraying. Error bars represent the SD (*n* = 3). Values followed by different letters are significantly different according to Tukey’s test (*P* < 0.05)

After 3.0 mg/L ozone water irrigation, the relative expression levels of chlorophyll synthesis-related genes *SlGLK1*, *SlGLK2*, and *SlDCL* in tomato leaves were significantly increased, reaching the maximum expression level at 12 h after treatment, with levels increased 59-, 23-, and 23-fold compared with the control. Among them, *SlGLK1* was increased the most (Fig. 8A).

After spraying 0.6 mg/L ozone water, the relative expression levels of *SlGLK1*, *SlGLK2*, and *SlDCL* were significantly upregulated and reached the maximum expression level at 12 h after treatment, with levels increased by 9-, 29-, and 37-fold compared with the control. Among them, *SlDCL* was increased the most, followed by *SlGLK2* and *SlGLK1* (Fig. 8B).

### Expression profiles of nitrogen absorption-related genes in tomato roots under optimal concentrations of ozone water irrigation and spraying

**Fig. 9:**
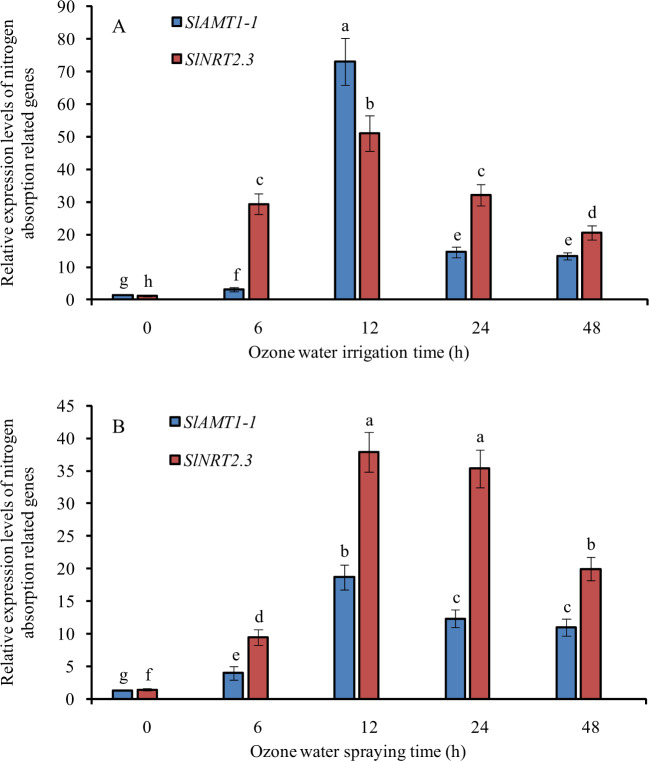
Relative expression of nitrogen absorption-related genes at 0, 6, 12, 24, and 48 h after ozone water treatments. **A** ozone water irrigation, **B** ozone water spraying. Error bars represent the SD (*n* = 3). Values followed by different letters are significantly different according to Tukey’s test (*P* < 0.05)

After 3.0 mg/L ozone water irrigation, the relative expression levels of the nitrogen absorption-related genes *SlAMT1-1* and *SlNRT2.3* in tomato roots were significantly increased, and the maximum expression level was reached at 12 h after treatment; levels were increased 50- and 42-fold compared with the control, and that of *SlAMT1-1* was increased more than that of *SlNRT2.3*, while after 6 h, 24 h and 48 h of treatment, that of *SlNRT2.3* was increased more than that of *SlAMT1-1* (Fig. 9A).

After spraying 0.6 mg/L ozone water, the relative expression levels of *SlAMT1-1* and *SlNRT2.3* were significantly upregulated and reached the maximum level at 12 h after treatment, with levels increased 13- and 25-fold compared with the control. At all ozone water spraying treatment times, the expression of *SlNRT2.3* was increased more than that of *SlAMT1-1* (Fig. 9B).

The results showed that the expression of antioxidant enzyme genes, chlorophyll synthesis-related genes, and nitrogen absorption-related genes in tomato seedlings could be upregulated by ozone water irrigation and spraying. Ozone water irrigation promoted the expression of genes in tomato seedlings better than ozone water spraying.

## Discussion

Membrane lipid peroxidation often occurs when plants are senescent or damaged under adverse conditions. MDA is the final decomposition product of membrane lipid peroxidation. MDA released from the production site on the membrane will interact with proteins and nucleic acids to modify their characteristics, thereby relaxing the bridge bonds between interacting molecules or inhibiting the synthesis of proteins. Therefore, the MDA content can reflect the extent to which plants are undergoing stress^18^. In other words, the lower the MDA content in plants is, the higher the relative tolerance of plants to oxidative stress. Therefore, the MDA content is a common indicator of plant stress tolerance. In this paper, compared with the control group, the MDA content in tomato seedling leaves was significantly decreased after ozone water irrigation and spraying, indicating that ozone water treatment can significantly improve the stress tolerance of tomato seedlings.

The antioxidant enzyme system is an important defense system in plants that can avoid oxidative damage and stabilize the intracellular environment. Superoxide dismutase (SOD), peroxidase (POD), and catalase (CAT) are important members of the antioxidant enzyme system. The activity of antioxidant enzymes is an important index of plant tolerance to stress^19,20^. In this paper, after ozone water treatment at the optimum concentration, the activities of SOD, POD, and CAT in tomato leaves were increased significantly, and the relative expression levels of *SlSOD*, *SlPOD*, *SlCAT2a*, and *SlCAT2b* were also increased significantly, indicating that ozone water treatment can significantly improve the stress tolerance of tomato seedlings, and the effect of ozone water irrigation is more obvious than that of leaf spraying. At the same time, we also noticed that the antioxidant enzyme activity of tomato leaves increased with increasing ozone water concentration before the ozone water concentration reached 3.0 mg/L, while the effect of 4.0 mg/L ozone water was significantly lower than that of 3.0 mg/L. It is reasonable to speculate that low-concentration ozone water treatment can significantly increase the antioxidant enzyme activity and improve the stress tolerance of plants, while high-concentration ozone water treatment, which has a certain toxic effect, can reduce the stress tolerance of plants. Therefore, the effect of 4.0 mg/L ozone water is not ideal.

The chlorophyll content can determine the photosynthetic rate and primary productivity of plants and is widely used in plant responses to environmental stress. The change in chlorophyll content will further affect the photosynthetic capacity of plants. Therefore, chlorophyll content can be used as an important diagnostic index for plant growth research, and the SPAD value has a significant positive correlation with the content of total chlorophyll, chlorophyll a, and chlorophyll b^21^. At the molecular level, Goldern-like (GLK) transcription factors belong to the GARP family and are widely identified in higher plants. Some GLK transcription factors act as regulators to promote chloroplast development^22^. *GLK1* encodes a disease defense-related protein in *Arabidopsis thaliana* and is resistant to *Fusarium graminearum*^23^. However, the absence of *GLK1* in mutant lines reduced the chlorophyll content and chloroplast development, which affected photosynthesis in plants^24^. GLK2 is an important transcription factor regulating chloroplast development in immature fruits. Previous studies showed that the shoulder of immature fruits with *SlGLK2* gene overexpression was dark green, and the chlorophyll content of the green shoulder at different developmental stages was significantly higher than that of the fruit bottom^25^. The highest expression of *DlDCL1* and *DlDCL2* in leaf and flower tissues of *Dimocarpus longan* indicated that *DlDCL1* and *DlDCL2* may be involved in photosynthesis and flower organ development^26^. Therefore, GLK1, GLK2, and DCL were proven to be positive regulators in chlorophyll synthesis pathways, possibly playing an important role in chloroplast development. By detecting the expression level of chlorophyll synthesis-related genes, we can more intuitively and scientifically reveal the molecular mechanism for improving photosynthetic capacity. In this paper, after ozone water irrigation and spraying, the SPAD values of leaves were significantly increased, and the expression level of chlorophyll biosynthesis-related genes (*SlGLK1*, *SlGLK2*, *SlDCL*) was upregulated, indicating that ozone water treatment can improve the chlorophyll content of leaves and promote photosynthesis of plants at physiological and molecular levels. It was further confirmed that these three genes were involved in the regulation of chlorophyll synthesis in tomato seedlings. It is reasonable to speculate that ozone water treatment can improve the disease tolerance of seedlings to a certain extent by upregulating the expression of *SlGLK1*, but the specific molecular mechanism is still unclear and needs to be further verified by subsequent experiments.

The N level also has a great influence on crop growth and yield. Topdressing with urea could increase the contents of chlorophyll, protein, and amino acids in leaves^27^. At present, the known gene systems related to nitrogen absorption mainly include the ammonium absorption gene *AMT*, nitrate absorption gene *NRT*, *GS* gene, and *GO/GAT* gene^28^. The main forms of nitrogen absorption by plants are ammonium nitrogen and nitrate nitrogen. Therefore, we selected *SlAMT1-1* (ammonium nitrogen absorption-related gene) and *SlNRT2.3* (nitrate nitrogen absorption-related gene) for detection^29,30^. Through the determination of nitrogen absorption-related genes, we can reasonably infer the main forms of nitrogen absorption by tomato seedlings after ozone water treatment. In this paper, after ozone water irrigation and spraying, the N content of tomato leaves was significantly increased, and the relative expression levels of *SlAMT1-1* and *SlNRT2.3* were significantly increased, suggesting that ozone water treatment is beneficial for promoting nitrogen absorption in tomato seedlings. In addition, the expression of *SlNRT2.3* increased more than that of *SlAMT1-1* after ozone water spraying, suggesting that the main form of nitrogen absorbed in tomato seedlings may be nitrate nitrogen under ozone water spraying.

The toxic effect of ozone on plants is related to its concentration. When the concentration of ozone in the environment is high enough, it will cause serious damage to clover leaves^31^. At the same time, high-concentration ozone treatment can reduce the contents of chlorophyll, carotenoids, and carbohydrates in citrus plants^32^ and decrease biomass production in mid-season soybean^33^. In recent years, ozone has become a research hotspot because of its high efficiency, lack of residues, strong oxidative abilities, and other advantages. Matlok et al. (2020)^34^ found that sorrel plants had greater antioxidant potential after 1 mg/L ozone treatment, while 5 mg/L ozone treatment for a short time could also increase the antioxidant activity of potato leaves^35^. In this paper, low-concentration ozone water irrigation and spraying could significantly increase the antioxidant enzyme activity of tomato leaves, in accordance with the above research^34^. The effect of ozone water irrigation at 4.0 mg/L was not as good as that at 3.0 mg/L, and the optimal spraying treatment concentration was not 0.8 mg/L but 0.6 mg/L, suggesting that high-concentration ozone water treatment has certain harmful effects on plants. Therefore, while making full use of the advantages of ozone water, it is correct to choose the optimal concentration of ozone water to avoid its toxic effects.

Based on the above analysis, ozone water irrigation and spray treatment can activate the complex stress response mechanism in tomato seedlings, leading to the upregulation of related genes, thus significantly improving the physiological characteristics and stress tolerance of tomato seedlings. The effects of ozone water irrigation were better than those of spraying.

## Conclusion

Under external stress, plants activate their own defense mechanisms to avoid risks. On the one hand, ozone water treatment at the optimal concentration can significantly reduce the MDA content in tomato leaves and significantly improve the antioxidant enzyme activity of tomato leaves; on the other hand, the expression of antioxidant enzyme genes in tomato leaves was also increased significantly. This result indicated that ozone water treatment can significantly improve the stress tolerance of tomato seedlings. Ozone water treatment can significantly increase the SPAD value and nitrogen content of tomato leaves and obviously promote the expression of chlorophyll synthesis-related genes and nitrogen absorption-related genes, which is beneficial for improving the photosynthetic capacity and yield of tomato. Considering the economy and production efficiency, the optimal concentration ozone water treatment should be adopted; moreover, the effect of ozone water irrigation was much better than that of leaf spraying.

## Materials and methods

### Material treatments

The test material was ‘Little Tom’ tomato seeds. The soil for tomato cultivation was the sandy loam soil of Wulong River Farm in Gaomi City, China, with an organic matter content of 3.82 mg/g and pH of 6.93. Tomato seedlings with a height of 10 cm grown for 10–15 days were selected for transplanting. Ozone water was prepared by an ozone water machine (PIONEEREP 6L2, Qingdao Pioneer Environmental Protection Technology Co., Ltd., China), and the concentration of ozone water was measured by a dissolved ozone detector (CLEAN OZS30, America). The treatment was completed within 5 min after the preparation of ozone water to ensure the application effect.

Ozone water irrigation treatment: the soil was irrigated with 1.0, 2.0, 3.0, and 4.0 mg/L ozone water before the tomato seedlings were transplanted and held under natural light, with a relative humidity of 40–50% and temperature of 22–25 °C. After 20 days of growth, tomato seedlings were irrigated with ozone water at the above concentration once again, and water irrigation was used as the control. The related physiological indexes were measured after 5 days. There were five plants in each treatment group, and three biological replicates were set for each treatment.

Ozone water spray treatment: the tomato seedling culture conditions were the same. After 15 days of seedling growth, 0.2, 0.4, 0.6, and 0.8 mg/L ozone water was sprayed onto the leaves. After 5 days, ozone water at the above concentration was sprayed onto the leaves again, and water was sprayed as the control. The related physiological indexes were measured after 5 days. There were five plants in each treatment group, and three biological replicates were set for each treatment.

The third compound leaf near the top was selected as the test material and was frozen in liquid nitrogen and stored at −80 °C for later use.

### Determination of physiological indexes

Determination of the malondialdehyde (MDA) content in tomato leaves was performed according to the method of Ma et al. (2020)^36^. The activity of superoxide dismutase (SOD) in leaves was measured by the NBT photoreduction method^37^, that of peroxidase (POD) in leaves was determined according to He et al. (2003)^38^, and that of catalase (CAT) in leaves was determined according to Verma and Mishra (2005)^39^. The soil and plant analysis development (SPAD) value and nitrogen (N) content of leaves were measured by a PJ-4N plant nutrition analyzer.

### Determination of the relative expression of genes

The optimal concentrations for the ozone water irrigation and spray treatments were obtained by analyzing the relevant physiological index data. The mature leaves and roots were taken at 0, 6, 12, 24, and 48 hour (h) after optimal concentration ozone water irrigation and spraying twice (20 days of tomato seedling growth). The leaf material was the same as above, and a 2-cm portion near the root tip was also selected. Materials were frozen in liquid nitrogen and stored at −80 °C for later use.

Total RNA was extracted from 100 mg tomato materials using TRIzol-A^+^ reagent (Tiangen, China). cDNA was synthesized with the PrimeScript™ RT reagent kit (Takara, Japan) according to the manual. Real-time quantitative PCR (RT-qPCR) was performed using ChamQ Universal SYBR qPCR Master Mix (Vazyme, China) with Applied Biosystems QuantStudio 5 (ABI, USA). The PCR system and protocol were set according to the SYBR mix instructions. The internal reference genes *Actin*-F (GGATCTTGCTGGTCGTGATTTTT) and *Actin*-R (TCTGGGCAACGGAACCTCTC) were selected to correct the expression of the target gene^40^. The relative expression of genes was calculated by the 2^−^^ΔΔCT^ method^41^. Three biological replicates were carried out for each RT-qPCR.

RT-qPCR primers, including those for *SlSOD*-F (TGTCACTACCCCTAAACCCCT), *SlSOD*-R (CCAGGAGCTCCATGTGTCAA), *SlPOD*-F (TCTACGACTTTTCATGCCCACA), *SlPOD*-R (GAGCAGCAAGGGCGATAATG), *SlCAT2a*-F (GTGGTGGTGATAAAGGAGGGT), *SlCAT2a*-R (AAGCTCCGCATAGCAAAAGG), *SlCAT2b*-F (TTGCAGGCTATGCTTTTTGGA), *SlCAT2b*-R (TAGCCTGGCCATCCTGCTTT), *SlGLK1*-F (GAATTTTCCGTAAGCAGTGGTG), *SlGLK1*-R (CTTCTCCTTGATTTAGGCTCGT), *SlGLK2*-F (ACAATCGGAGGCGGAGGA), *SlGLK2*-R (CAAGGAGTGCCTGGTACAAGAG), *SlDCL*-F (CCGCAAGGATGGTGAAACA), *SlDCL*-R (TCCGCTTCCGAAAATGCC), *SlAMT1*-F (CTCCGTTCCTCGGTCCTAAC), *SlAMT1*-R (CATTAGACGGACCACCCCAAG), *SlNRT2.3*-F (TGTACACTTCCAGTAATGTTAGTT), and *SlNRT2.3*-R (GGTACCCAGACGCGATTTGGTGTTA), were designed by Primer 5.0 software.

### Data processing

Microsoft Office Excel 2007 was used to process and analyze the data, and the significance of differences was analyzed by group data *t*-test^42^.
